# 

**Published:** 1896-05

**Authors:** 


### TABLE OF CONTENTS.

ORIGINAL COMMUNICATIONS. PAGE

Dental and Facial Evidences of Constitutional Defect. By Eugene S. Talbot, M.D., D.D.S., Chicago, Ill. . . . . . . . . . . . . . . . . . . 261

Notes on Formic Aldehyde. By J. Morgan Howe, M.D., M.D.S., New York . . . . . . . . . . . . . . . . . . . . . . . . . . . . . 286

A Talk on Materia Medica. By Dr. E. C. Briggs, Boston, Mass. . . . . 294

REPORTS OF SOCIETY MEETINGS.

The New York Institute of Stomatology . . . . . . . . . . . . . . . . . 300

American Academy of Dental Science . . . . . . . . . . . . . . . . . . 310

Academy of Stomatology . . . . . . . . . . . . . . . . . . . . . . . . 314

EDITORIAL.

The Art of Professional Teaching . . . . . . . . . . . . . . . . . . . 324

Personal Explanation . . . . . . . . . . . . . . . . . . . . . . . . . 327

Delayed Matter . . . . . . . . . . . . . . . . . . . . . . . . . . . . 327

BIBLIOGRAPHY . . . . . . . . . . . . . . . . . . . . . . . . . . . . 327

OBITUARY.

Resolutions of Respect—Dr. W. H. Dwinelle . . . . . . . . . . . . . . 328

DOMESTIC CORRESPONDENCE.

Louisville Odontological Society . . . . . . . . . . . . . . . . . . . 330

An Important Correction . . . . . . . . . . . . . . . . . . . . . . . 330

NOTES AND COMMENTS . . . . . . . . . . . . . . . . . . . . . . . . 331

CURRENT NEWS.

The Golden Anniversary . . . . . . . . . . . . . . . . . . . . . . . . 334

American Medical Association . . . . . . . . . . . . . . . . . . . . . 337

Louisiana State Dental Society . . . . . . . . . . . . . . . . . . . . 338

Dental Society of the State of New York . . . . . . . . . . . . . . . . 338

New Orleans Academy of Stomatology . . . . . . . . . . . . . . . . . . 339

SELECTIONS.

Parachlorophenol . . . . . . . . . . . . . . . . . . . . . . . . . . . 339

Disinfectants . . . . . . . . . . . . . . . . . . . . . . . . . . . . . 339

A New Hæmostatic . . . . . . . . . . . . . . . . . . . . . . . . . . . 340

